# Detection of Colorectal Serrated Polyps by Stool DNA Testing: Comparison with Fecal Immunochemical Testing for Occult Blood (FIT)

**DOI:** 10.1371/journal.pone.0085659

**Published:** 2014-01-20

**Authors:** Russell I. Heigh, Tracy C. Yab, William R. Taylor, Fareeda T. N. Hussain, Thomas C. Smyrk, Douglas W. Mahoney, Michael J. Domanico, Barry M. Berger, Graham P. Lidgard, David A. Ahlquist

**Affiliations:** 1 Division of Gastroenterology at Mayo Clinic, Scottsdale, Arizona, United States of America; 2 Division of Gastroenterology and Hepatology at Mayo Clinic, Rochester, Minnesota, United States of America; 3 Mayo Medical School, Rochester, Minnesota, United States of America; 4 Department of Laboratory Medicine and Pathology, Mayo Clinic, Rochester Minnesota, United States of America; 5 Department of Biomedical Statistics and Informatics, Mayo Clinic, Rochester Minnesota, United States of America; 6 Exact Sciences Corporation, Madison, Wisconsin, United States of America; University of Bari & Consorzio Mario Negri Sud, Italy

## Abstract

**Objectives:**

Precursors to 1/3 of colorectal cancer (CRC), serrated polyps have been under-detected by screening due to their inconspicuous, non-hemorrhagic, and proximal nature. A new multi-target stool DNA test (multi-target sDNA) shows high sensitivity for both CRC and advanced adenomas. Screen detection of serrated polyps by this approach requires further validation. We sought to assess and compare noninvasive detection of sessile serrated polyps (SSP) ≥1 cm by sDNA and an occult blood fecal immunochemical test (FIT).

**Methods:**

In a blinded prospective study, a single stool sample used for both tests was collected from 456 asymptomatic adults prior to screening or surveillance colonoscopy (criterion standard). All 29 patients with SSP≥1 cm were included as cases and all 232 with no neoplastic findings as controls. Buffered stool samples were processed and frozen on receipt; Exact Sciences performed sDNA in batches using optimized analytical methods. The sDNA multi-marker panel targets methylated *BMP3* (m*BMP3*) and *NDRG4*, mutant *KRAS*, *β-actin*, and hemoglobin. FIT (Polymedco OC-FIT Check) was performed in separate lab ≤2 days post defecation and evaluated at cutoffs of 50 (FIT-50) and 100 ng/ml (FIT-100).

**Results:**

Median ages: cases 61 (range 57–77), controls 62 (52–70), p = NS. Women comprised 59% and 51%, p = NS, respectively. SSP median size was 1.2 cm (1–3 cm), 93% were proximal, and 64% had synchronous diminutive polyps. Among multi-target sDNA markers, m*BMP3* proved highly discriminant for detection of SSP≥1 cm (AUC = 0.87, p<0.00001); other DNA markers provided no incremental sensitivity. Hemoglobin alone showed no discrimination (AUC = 0.50, p = NS). At matched specificities, detection of SSP≥1 cm by stool m*BMP3* was significantly greater than by FIT-50 (66% vs 10%, p = 0.0003) or FIT-100 (63% vs 0%, p<0.0001).

**Conclusions:**

In a screening and surveillance setting, SSP≥1 cm can be detected noninvasively by stool assay of exfoliated DNA markers, especially m*BMP3*. FIT appears to have no value in SSP detection.

## Introduction

Prevention of colorectal cancer (CRC) by screening rests on effective detection of the critical precursor lesions. Based on the assumption that CRC evolves primarily via the established adenoma-to-carcinoma pathway [1], screening has historically focused on detecting adenomas as the exclusive precursor lesion. However, it has become increasingly apparent in recent years that many CRCs arise from serrated polyps, which differ biologically and clinically from conventional adenomas [2], [3].

Serrated polyps, often classified in the past as innocuous hyperplastic polyps and disregarded, are now thought to represent the essential progenitor of up to 1/3 of all CRC [4]–[6]. Sessile serrated adenomas or polyps (SSPs) represent the subset of serrated polyps at greatest risk of CRC progression and may grow more rapidly than conventional adenomas [4]–[6]. SSPs are typically right-sided, often flat and endoscopically inconspicuous, and relatively more common in women and the elderly [6]–[9]. Unlike conventional adenomas, SSPs are commonly associated with *BRAF* mutations [3], [10]; while both SSPs and conventional adenomas frequently harbor aberrantly methylated genes [6], [11], [12]. SSPs that progress often acquire dysplasia, a transition accompanied by microsatellite instability [4], [8], [13], [14]; and remnants of SSP tissue can be demonstrated at margins of right-sided CRC [13], [15].

Data on SSP detection by conventional screening tools are limited, but indirect evidence suggests that their effectiveness is suboptimal. Despite trends of increasing polypectomy rates and reductions in left-sided CRC, population-based studies show that intensified screening has had proportionately less effect on the incidence of right-sided CRC [16]. Sigmoidoscopy is inherently limited to visualization of the left colorectum, and its programmatic application has little impact on proximal CRC [17], [18]. Fecal blood testing, whether by guaiac [19], [20] or immunochemical [21], [22] methods, exhibits low detection rates for adenomas, especially proximal ones; large screening studies reveal that guaiac-type fecal blood testing has had negligible or minimal effect on CRC incidence [20], [21]; and it is not known if they can detect SSPs which are largely non-hemorrhagic. Even colonoscopy appears to detect right-sided precursor lesions less well than left-sided ones, despite its capacity to directly inspect the entire colorectum [23]–[25]. The relatively lower effectiveness of current screening approaches to affect proximal colon cancer may relate to the nature of right-sided precursor lesions and to our difficulties in detecting them with current tools.

Stool DNA testing represents a biologically rational candidate approach to the screen detection of SSPs that merits further evaluation. With the improved analytical sensitivity of next generation assays, stool DNA testing has been shown to detect advanced adenomas at high rates [26]–[29], and neoplasm site does not appear to affect test sensitivity [20], [27]. Preliminary reports on selected patients suggest that stool DNA testing detects large SSPs at sensitivities comparable to those for adenomas. In one small study using archived stool specimens [30], both mutant *BRAF* and selected methylated gene markers were elevated in stools from patients harboring large SSPs. In the other preliminary report [31], an optimized pre-commercial multi-marker stool DNA test (sDNA), which targets aberrantly methylated *BMP3* and *NDRG4* genes, detected 60% of SSP≥1 cm at 90% specificity.

In the present prospective study, we sought to assess the noninvasive detection of SSP by stool DNA testing in asymptomatic persons undergoing screening or surveillance colonoscopic examination of the colon. Our specific aims were to 1) evaluate the performance of a pre-commercial multi-target stool DNA test (multi-target sDNA) for detection of SSP≥1 cm, 2) determine which multi-target sDNA markers contribute most to SSP detection based on stool and tissue analyses, and 3) compare SSP detection rates by assay of exfoliated markers using stool DNA testing with those by assay of occult bleeding using a quantitative fecal immunochemical test (FIT). We hypothesized that asymptomatic SSP lesions ≥1 cm would exfoliate markers at rates sufficient to be detected by sDNA testing but would rarely bleed and not be meaningfully detected by FIT.

## Methods

This blinded cross-sectional study was approved by the Mayo Clinic Institutional Review Board on September 7, 2010 and conducted at Mayo Clinic facilities in Scottsdale AZ and Rochester MN. All stool and tissue assays were performed by technicians unaware of clinical source of samples. The Mayo Clinic Institutional Review Board deemed this study of de-identified biospecimens to be of minimal risk. Using a form approved by the Mayo Clinic Institutional Review Board, written informed consent was obtained prior to specimen collection.

### Participants in Stool Study

Participants comprised consenting asymptomatic adults scheduled during 2011–2012 for a screening or polyp surveillance colonoscopy, which served as the criterion standard. Patients were excluded if they had (1) a prior colorectal resection, (2) inflammatory bowel disease, Lynch syndrome, familial adenomatous polyposis, or other high risk conditions for CRC, (3) colonoscopy that was incomplete or associated with a poor preparation, or (4) a prior screening examination was done within 5 years. All patients found to have a SSP≥1 cm without a synchronous advanced adenoma or CRC were designated as cases. The presence of synchronous small or diminutive polyps did not exclude cases; rather, stratified stool data analyses were performed on patient subsets with and without synchronous small or diminutive polyps. Data were not included on isolated lesions smaller than 1 cm in this proof-of-concept study. All patients found to be free of colorectal polyps or tumors were designated as controls.

### Stool Collection and Storage

A single stool from each participant was prospectively collected prior to cathartic cleansing for colonoscopy using a bucket container mounted to the toilet seat. Patients sampled stools for FIT analyses using probe devices; and then added a preservative solution to the whole stool and promptly mailed the sealed container along with FIT tubes to the processing laboratories, as described [28].

Upon receipt, stools were homogenized, aliquoted, and frozen at −80°C for subsequent batch performance of the multi-marker sDNA assay. The commercial FIT assay was performed upon receipt (see below). Stools received >3 days after defecation were disqualified and not tested.

### Stool Assay Methods

#### Stool DNA tests

Stool processing, assay methods, and primer sequences have been described in detail [28], [29], [31], [32]. This pre-commercial multi-target sDNA assay was performed at Exact Sciences (Madison WI) and included the following recent innovations: automation, direct gene capture from fecal supernatant, an optimized rapid bisulfite treatment process, a panel of broadly informative DNA markers (m*BMP3*, *mNDRG4*, mutant *KRAS,* and *β-actin*) assayed in multi-plex by the analytically-sensitive quantitative allele-specific real-time target and signal amplification (QuARTS) method, a proprietary quantitative fecal hemoglobin assay, and use of a logistic regression model for analysis. Results for the sDNA test were designated as “positive” or “negative” by the manufacturer based on their pre-established logistic algorithm. Individual tumor markers from the multi-target sDNA panel were normalized to stool *β-actin* (a marker of total human DNA) and evaluated separately as well; specificity cutoffs were selected to match those of FIT in the comparison studies.

#### Fecal Immunochemical Test for occult blood (FIT)

A quantitative commercially available FIT (OC-FIT CHEK, Polymedco, NY) was performed at Mayo Clinic using an automated reading device. FIT results were evaluated at the manufacturer’s recommended specificity cutoff of 100 ng hemoglobin/ml buffer (FIT-100) and also at a cutoff of 50 ng/ml (FIT-50).

#### Tissue analyses

To determine whether SSP lesions represent the likely origin of methylated markers in stool, a blinded independent tissue study was performed on SSP lesions ≥1 cm colonoscopically-removed from 20 unique case patients [median age 61 (range 30–83), 68% women] and on normal colon mucosa biopsies from 20 unique control patients without visible colorectal lesions [median age 54 (range 30–81), 70% women]. Following micro-dissection of paraffin or frozen tissue slides, DNA was extracted in usual fashion. Bisulfite treatment and marker assay methods were as described above for stool. Quantitative levels were normalized to *β-actin.*

### Statistical Analysis

Associations between test positivity and clinical characteristics were assessed using the Chi-square test. Comparison of sensitivities between tests was done at matched specificities using McNemar’s test for paired proportion. The non-parametric Wilcoxon Rank Sums Test was used to test the association between continuous marker values and clinical characteristics. The discriminant accuracy of each marker was estimated as the area under the ROC curve (AUC) [33]. Linear combination of markers in the multi-target sDNA test for the prediction of disease status was assessed using logistic regression. To avoid over fitting of the data, the final set of predictors used within the logistic regression model was determined from fitting an Elastic Net regression model with all the markers; and the most discriminant marker or set of markers was selected to have the lowest cross-validated prediction error [34].

## Results

### Patient and Lesion Characteristics for Stool Study

From 456 asymptomatic patients undergoing screening or surveillance colonoscopy, we identified 29 individuals with SSP≥1 cm who served as cases and 232 free of polyps who served as controls. Age and sex distributions were similar between groups (Table 1).

**Table 1:** Patient and Lesion Characteristics for Stool Study.

	SSP Cases (29)^a^	Normal Controls(232)^b^
Patient Demographics		
Age in years, median (range)	62 (57–77)	61 (52–70)
Sex, % women	59	52
SSP Features		
Size in cm, median (range)	1.4 (1.0–3.0)	–
Right sided, %^c^	93	–
Dysplasia present, %	3	–
Synchronous small polyps, %^d^	64	–

Abbreviation: –, not applicable.

^a^ Cases comprised patients with at least one SSP (sessile serrated polyp) ≥1 cm found on screening or surveillance colonoscopy and without synchronous advanced adenomas or CRC.

^b^ Control patients had no pathology (no CRC, colorectal polyps, hemorrhagic lesions, or inflammation) on screening or surveillance colonoscopy.

^c^ Right-sided location was defined as proximal to the splenic flexure.

^d^ Patients found to harbor synchronous polyps (adenomatous or serrated) <1 cm in size were included as cases.

In cases, median SSP size was 1.2 cm (range 1–3), 28/29 (93%) were located proximal to the splenic flexure, and only one SSP contained dysplasia (Table 1). Synchronous small or diminutive polyps were present in 64% of cases.

### SSP Detection by the Multi-target Stool DNA Test

#### Test accuracy

Using the manufacturer’s current cutoff, the sensitivity of the sDNA test for detection of SSP≥1 cm was 55% (95% CI: 36–74) and the specificity was 91% (95% CI: 87–94).

#### Effect of covariates

The SSP detection rate did not differ significantly between case subsets with and without synchronous small or diminutive polyps; sensitivities were 60% and 50%, respectively (p = 0.4). Within the narrow size range of SSP cases identified, the detection rate was 55% for the 11 patients with lesions 1–1.4 cm and 53% for those 17 with lesions 1.5–3 cm, p = 0.9. Neither age nor sex influenced SSP detection rates (data not shown).

#### Contribution of component markers to SSP detection

Among the multi-target sDNA markers, *mBMP3* proved most discriminant for SSP detection, with an AUC of 0.87 (95% CI: 0.80–0.95), p<0.0001. The other informative DNA markers, *mNDRG4* (AUC 0.79; 95% CI; 0.70–0.88; p<0.0001) and mutant *KRAS* (AUC 0.64; 95% CI: 0.53–0.75; p = 0.0068), did not provide statistically significant incremental sensitivity in this study above that provided by *mBMP3*. Of note, fecal hemoglobin alone showed no discrimination (AUC = 0.50; 95% CI: 0.40–0.61; p = 0.4724).

### Tissue Confirmation of SSP Discrimination by Methylation Markers

**Figure 1:**
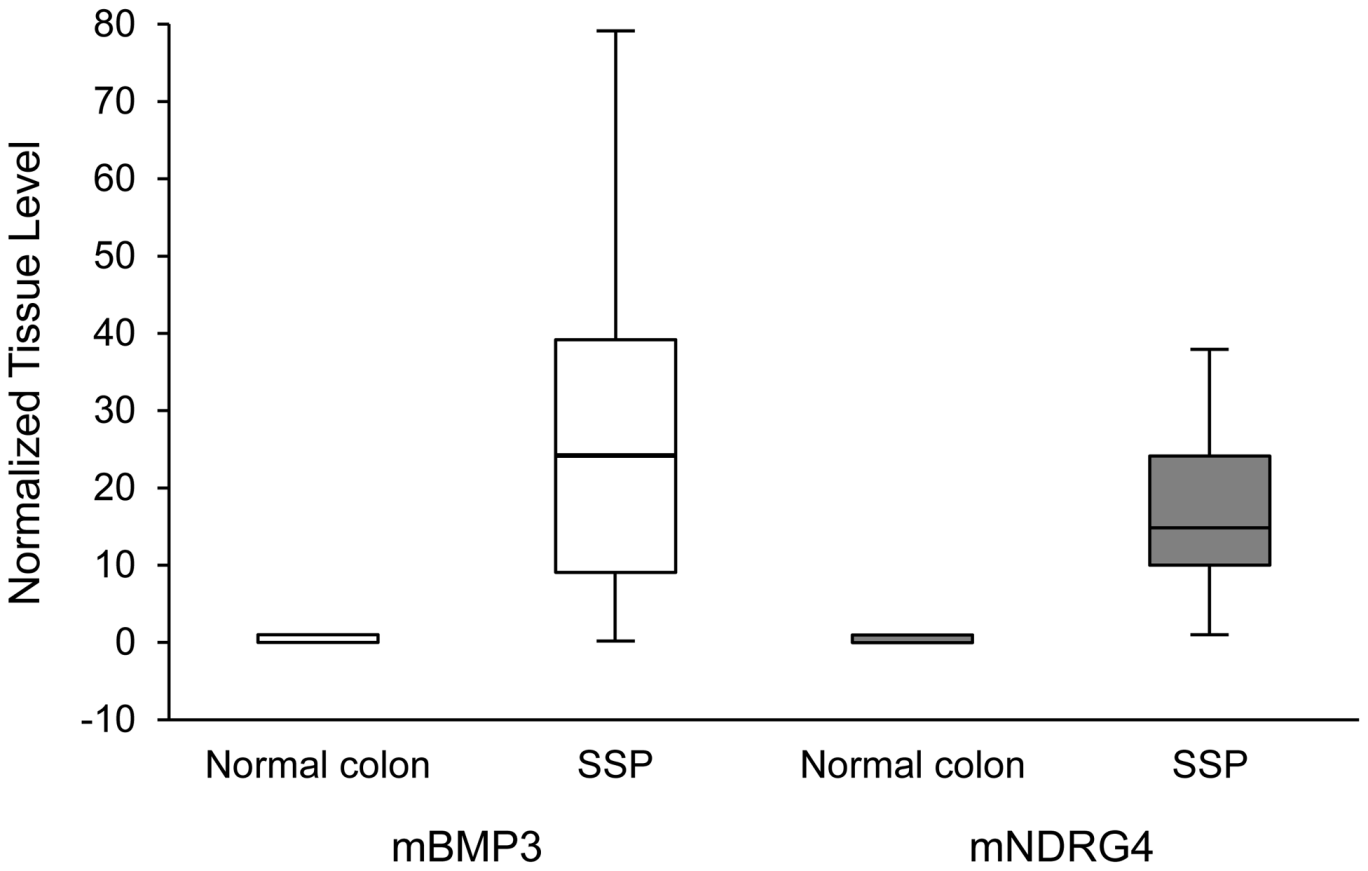
Tissue levels of aberrantly methylated genes. Tissue levels of methylated *BMP3 (mBMP3)* and *NDRG4 (mNDRG4)* genes are compared in normal colorectal mucosa, n = 20 unique control patients, and sessile serrated polyps (SSP), n = 20 unique case patients. Marker levels are normalized to *β-actin* (a marker of total human DNA) and expressed in relative units. Levels were substantially and significantly higher in SSP than normal colon for both *mBMP3* (p<0.0001) and *mNDRG4* (p<0.0001).

At the tissue level, each methylated marker alone discriminated SSP from normal colon mucosa almost completely (Figure 1). The median *mBMP3* level in SSP lesions (24 relative units (range 0–78) was >200 times greater than in normal colon mucosa (0.11 (range 0–0.11), p<0.0001. The median *mNDRG4* level in SSP lesions (15 (range 0.9–47)) was >70 times higher than in normal colon mucosa (0.21 (0–0.8)), p<0.0001.

### Comparison of Stool DNA Testing and FIT for SSP Detection

**Figure 2:**
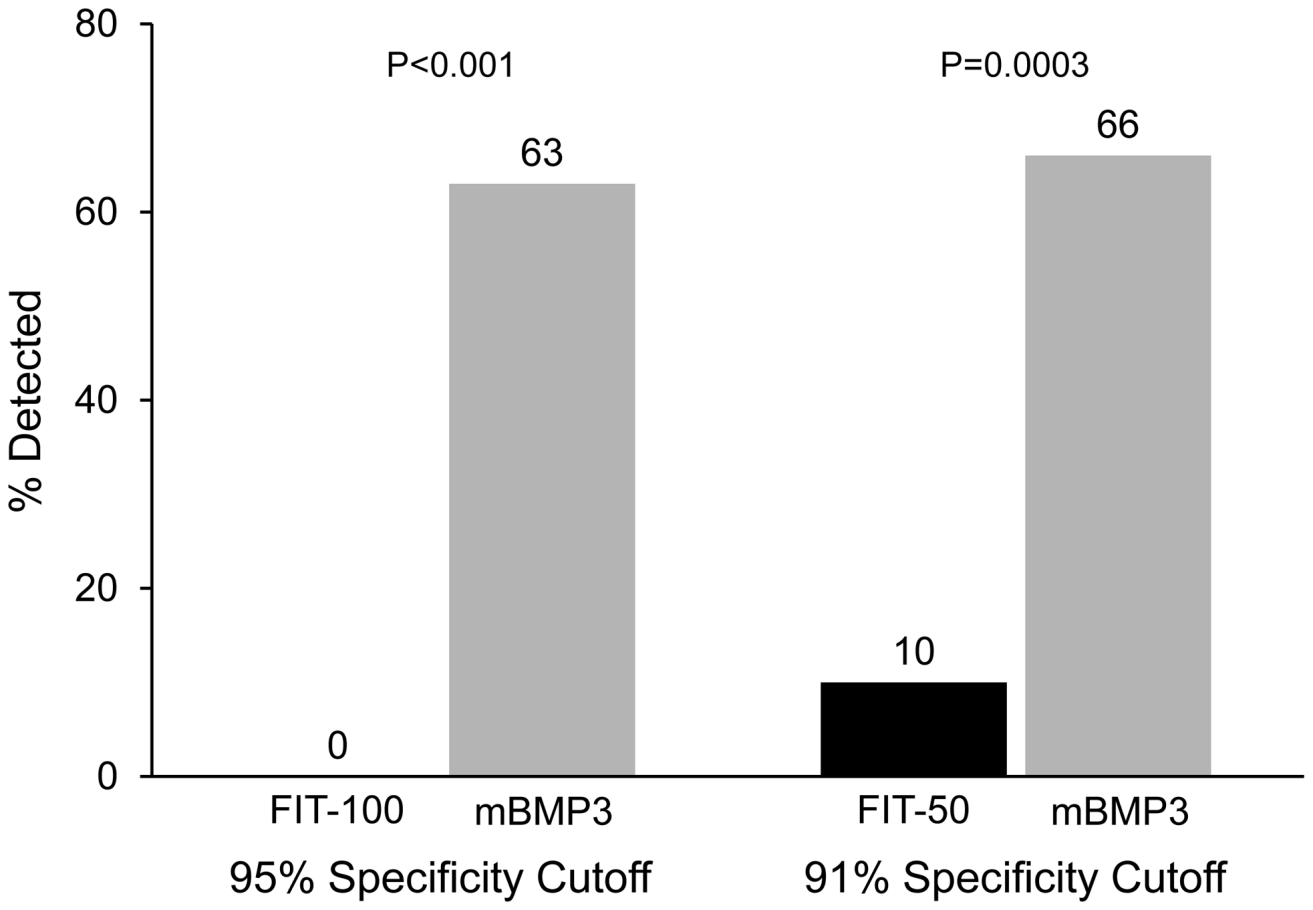
Detection of SSP by stool assay of *mBMP3* and by fecal immunochemical testing (FIT). FIT sensitivity was evaluated at the conventional cutoff of 100/ml buffer (FIT-100) and at 50 ng/ml (FIT-50). Specificity cutoffs for stool DNA marker *mBMP3* were selected to match those for both FIT-100 and FIT-50 so that sensitivities could be most meaningfully compared.

Stool assay of *mBMP3* (an exfoliated DNA marker) was selected for comparison against FIT, which measures fecal hemoglobin (an occult bleeding marker), to best evaluate the diagnostic yield of these markers that enter stool via biologically distinct mechanisms. At matched specificities, stool assay of *mBMP3* was substantially and significantly more sensitive for detection of SSP≥1 cm than FIT (Figure 2). At 95% specificity cutoffs, stool assay of *mBMP3* detected 63% of SSP lesions compared to 0% by FIT-100, p<0.0001; at 91% specificities, stool assay of *mBMP3* detected 66% compared to 10% by FIT-50, p = 0.0003.

## Discussion

Based on this prospective case-control study on asymptomatic patients undergoing screening or surveillance colonoscopy, we demonstrate that stool DNA testing represents a feasible approach to the noninvasive detection of SSP≥1 cm. Stool assay of *mBMP3*, a component of the multi-target stool DNA test, primarily accounted for the high SSP detection rates. Given our prior demonstrations that advanced adenomas can be detected at high rates by a next generation multi-target stool DNA test [26]–[29], [31], [32], these new findings suggest broadened value of this tool for CRC prevention through the detection of critical precursor lesions from both major molecular pathways of carcinogenesis.

In contrast to stool DNA testing, FIT essentially failed to detect SSPs. This finding is perhaps not surprising given the biology of these precursor lesions. SSPs are typically sessile or flat, non-ulcerated, and without hemorrhagic features [5], [6], [9]. In contrast, while SSPs may not bleed, our findings demonstrate that they do exfoliate at rates sufficient enough to allow their noninvasive detection by assay of altered DNA in stool.

The specific point sensitivities by sDNA testing for detection of SSP≥1 cm that we observed could translate to substantial practical value. Repeat screening with a test having a point sensitivity of 55–67% yields a potential program sensitivity exceeding 90% after the third screening round [35]. These programatic detection rates may compare favorably with conventional colonoscopy done every 10 years, particularly given the reported wide range in operator variation with colonoscopy [36].

Accurate detection of gastrointestinal neoplasms by stool assay of exfoliated markers requires high analytical sensitivity and discriminant markers. The QuARTS assay method used in this study achieves more than 100-fold higher sensitivity than earlier generation stool DNA assays [26]-[29], [31], [32] and, as we observed in this study, provides the critical analytical power to detect low-abundance DNA markers exfoliated into stool from SSPs. SSP detection by the multi-target stool DNA test was primarily accounted for by *mBMP3*. Consistent with the stool observations, we found in our tissue study that *mBMP3* levels highly discriminated SSP from normal colon mucosa. Perhaps because of the low background levels of *mBMP3* in normal control stools, assay of this marker alone achieved slightly higher SSP detection rates than did assay of the full marker panel in this study.

Strengths of this study included prospective stool collections from well-characterized patients undergoing colonoscopy for average risk screening or polyp surveillance, a blinded design, state-of-the-art assay technology, and use of discriminant methylation markers. Study limitations comprised the relatively narrow and small SSP size range, as the median size was only 1.2 cm, and a paucity of lesions containing dysplasia which likely represent those at greatest risk of progression. Despite these SSP characteristics, the majority were detected by the multi-target sDNA test. Furthermore, the observed differences in SSP detection rates between the stool DNA testing and FIT were substantial and highly significant. Sample size in this study did not permit robust covariate analyses.

We demonstrate proof-of-concept for SSP detection by stool DNA testing. Clinical applications of these findings can be considered and further explored. Incorporating markers specific for SSP into stool DNA tests designed for general CRC screening has potential to expand the effectiveness of this approach for CRC prevention, as both major types of precursor lesions could be targeted. Furthermore, given that colonoscopy has had a proportionately lower impact on incidence and mortality from right-sided than left-sided CRC [23]–[25] and that the majority of interval CRC cases are right-sided [37], a rationale could be made for the complementary use of stool DNA testing between screening colonoscopies, which are recommended at a frequency of every 10 years. While an economic analysis of an enhanced colorectal neoplasia detection strategy is beyond the scope of this study, once accurate assumptions of costs and test performance characteristics are available, modeling of new approaches will be instructive. The multi-target sDNA test is currently being evaluated in a prospective study involving >10,000 patients from the screen setting using colonoscopy as the criterion standard (the DEEP-C Study), and data from this large study should provide important and robust validation of this noninvasive screening approach.
